# Trends in oral corticosteroids use in severe asthma: a 14-year population-based study

**DOI:** 10.1186/s12931-021-01696-x

**Published:** 2021-04-09

**Authors:** Mohsen Sadatsafavi, Amir Khakban, Hamid Tavakoli, Solmaz Ehteshami-Afshar, Larry D. Lynd, J. Mark FitzGerald

**Affiliations:** 1 Respiratory Evaluation Sciences Program, Faculty of Pharmaceutical Sciences, The University of British Columbia, Vancouver, Canada; 2 Collaboration for Outcomes Research and Evaluation, Faculty of Pharmaceutical Sciences, The University of British Columbia, Vancouver, Canada; 3 Division of Respiratory Medicine, Department of Medicine, The University of British Columbia, Vancouver, Canada

**Keywords:** Asthma, Asthma/*drug therapy, Drug Prescriptions/*statistics & numerical data, Corticosteroids, Cohort Studies

## Abstract

**Background:**

Oral corticosteroids are important components of pharmacotherapy in severe asthma. Our objective was to describe the extent, trends, and factors associated with exposure to oral corticosteroids (OCS) in a severe asthma cohort.

**Methods:**

We used administrative health databases of British Columbia, Canada (2000–2014) and validated algorithms to retrospectively create a cohort of severe asthma patients. Exposure to OCS within each year of follow-up was measured in two ways: maintenance use as receiving on average ≥ 2.5 mg/day (prednisone-equivalent) OCS, and episodic use as the number of distinct episodes of OCS exposure for up to 14 days. Trends and factors associated with exposure on three time axes (calendar year, age, and time since diagnosis) were evaluated using Poisson regression.

**Results:**

21,144 patients (55.4% female; mean entry age 28.7) contributed 40,803 follow-up years, in 8.2% of which OCS was used as maintenance therapy. Maintenance OCS use declined by 3.8%/calendar year (p < 0.001). The average number of episodes of OCS use was 0.89/year, which increased by 1.1%/calendar year (p < 0.001). Trends remained significant for both exposure types in adjusted analyses. Both maintenance and episodic use increased by age and time since diagnosis.

**Conclusions:**

This population-based study documented a secular downward trend in maintenance OCS use in a period before widespread use of biologics. This might have been responsible for a higher rate of exacerbations that required episodic OCS therapy. Such trends in OCS use might be due to changes in the epidemiology of severe asthma, or changes in patient and provider preferences over time.

**Supplementary Information:**

The online version contains supplementary material available at 10.1186/s12931-021-01696-x.

## Background

Around 2.4% to 4% of patients with asthma suffer from a severe or difficult-to-control phenotype at any given time[1, 2], and 1 in 3 asthma patients shows at least one feature compatible with severe asthma in the previous twelve months[3]. Compared to the general asthma population, these patients suffer from a higher rate of morbidity due to frequent exacerbations and symptom burden [2, 4]. Systemic corticosteroids are commonly used for the management of severe asthma, either episodically for moderate-to-severe exacerbations, or chronically as maintenance therapy in patients who do not respond satisfactorily to inhaled therapies [5]. As a result, patients with persistent severe asthma often suffer from systemic side effects of corticosteroids such as diabetes, hypertension, glaucoma, osteoporosis, and mood disorders [6–9].

Studying the patterns and trends in the use of systemic corticosteroids is timely. In recent years, many novel biologics with marked effects in patients with severe asthma have been or are being developed. The hallmark of these studies has been a reduction in exacerbation frequency and the associated episodic oral corticosteroid (OCS) use [10, 11]. Such therapies have also resulted in lower dependence on maintenance OCS therapy in many patients [12, 13]. As such, the benefit of such therapies is not only due to a reduction in the incidence of exacerbations and improvement in symptom control, but also in the prevention of corticosteroid-related adverse events. The far-reaching implications of lower exposure to systemic corticosteroids necessitate a more precise estimation of the current and future patterns of systemic corticosteroids use in severe asthma.

While previous studies have evaluated factors associated with the risk of corticosteroid use [9, 14] and outcomes of exposure to corticosteroids in severe asthma [6–9], there is a lack of evidence on the extent and trends of maintenance and episodic use of OCS in patients with severe asthma. Population-based databases are uniquely positioned to fill this evidence gap. Such databases are not affected by the self-selection of individuals into private insurance plans, or the strict inclusion criteria of clinical studies, thus making them suitable for the analysis of population-level trends. The aim of the present study was to use data for a well-defined population of an entire geographic region to provide estimates of the extent and trends of exposure to OCS in patients with severe asthma, and to evaluate factors associated with OCS use.

## Methods

### Study population

For this descriptive retrospective cohort study, we used administrative health databases of the province of British Columbia (BC), Canada (population 4.6 million as of 2014) between 1997 and 2014. The public healthcare system in BC covers all legal residents and as such, the administrative health data are representative of the entire population. The first three years of data were not used in order to allow sufficient time for asthma patients to be captured in longitudinal records. In addition, due to the one-year requirement for the case definition (see below), not all asthma cases were captured during the last year of observation (2014), thus the analysis of trends was restricted to the 2000–2013 period. We had access to registration and demographic databases that capture basic socio-demographic information and the registration status of individuals in the provincial healthcare plan [15], the Vital Statistics database that captures the dates of birth and death [16], the Discharge Abstract Database that captures periods of hospital admissions and discharge diagnoses based on International Classification of Diseases (ICD) codes [17], the Medical Services Plan database which includes records of all outpatient services use and associated ICD-codes [18] and PharmaNET, a dataset that captures all medication dispensation records regardless of the type of third-party insurance use [19]. All inferences, opinions and conclusions drawn in this research are those of the authors and do not reflect the opinions or policies of the Data Steward(s). The University of British Columbia’s Clinical Research Ethics Board approved this research (application H15-00062).

### Asthma case definition

We applied a validated case definition to create a cohort of patients with diagnosed asthma[20], which is based on satisfying at least one of the three following criteria during any rolling 12-month window in patients who were 14 to 45 years of age: (1) one hospitalisation with the main discharge ICD diagnosis code of asthma, or (2) two physician visits on different dates with an ICD code for asthma, or (3) filled prescriptions on different dates for at least three asthma-related medications (according to a pre-specified list, Additional file 1: Table S1). ICD, 9th revision code of 493.XX and 10th revision codes of J45 or J46 were used for asthma. The lower age limit applied in this study is a widely used cut-off for the distinction between paediatric and adolescent/adult asthma. The upper age limit was chosen to prevent the inclusion of patients with permanent airflow obstruction without asthma who generally use similar inhaled medications. However, once the case definition was met, the patients could remain in the dataset regardless of age (given the 14 years of data, this would result in a maximum age of 58 years in the study).

Within this asthma cohort, the ‘index date’ was defined for each patient as the first date of any asthma-related resource use and marked the beginning of follow-up. Follow-up was ended at the earliest of the last resource use of any type, de-registration from the system, or death. The follow-up time was divided into adjacent 12-month periods, during which asthma severity and OCS exposures were assessed. If the last period was < 12 months in length it was removed from the subsequent analyses. Only patients with at least one follow-up period associated with severe asthma, as defined below, remained in the final cohort.

### Assessing asthma severity

Each follow-up period for each patient was independently labelled as severe or non-severe asthma based on the patterns of asthma-related resource use according to a validated algorithm [21]. The algorithm has been developed using Canadian administrative databases and has been validated against the Canadian consensus guidelines for the definition of asthma severity. In general, this definition is concordant with the definition proposed by the joint European Respiratory Society/American Thoracic Society Task Force on severe asthma [22], is similar to other claims-based definitions [23], and has been used in multiple previous studies [24–26]. In summary, healthcare records in a patient-year were considered as being compatible with severe asthma based on a combination of high doses of inhaled corticosteroid dispensed (defined as > 1000 μg prednisone-equivalent), frequent filled prescriptions of inhaled short-acting beta agonists (SABA), the filled prescriptions of other controller therapies, and markers of exacerbations such as emergency department visits and hospital admissions.

### Outcomes

Two indices pertinent to OCS use were defined and analysed as co-primary outcomes. First, a follow-up period was considered as associated with maintenance OCS use (a binary variable) if the average prednisone-adjusted daily dose of OCS during the 12-month period was 2.5 mg or more (this is equivalent to 6 months of exposure to 5 mg prednisone, as used in previous studies [9]). Second, episodic OCS use (a count variable) was calculated for each follow-up period as the number of distinct two-week episodes of OCS use, which were at least 7 days apart from any preceding or succeeding OCS use record. We did attempt to separate OCS use due to asthma versus other chronic conditions, given the inaccuracy of attributing a particular prescription to asthma or other conditions. Instead, in addition to documenting raw trends, we performed sensitivity analyses that adjusted for the presence of co-existing conditions.

### Statistical analysis

Trends over three time axes were evaluated: calendar year, age, and duration of asthma. In all analyses, only follow-up periods that were labelled as severe asthma were included. Each follow-up period was assigned to a calendar year or year of age according to the starting date of the period. For the analysis of trends over the time-course of severe asthma, a subgroup of incident severe asthma was defined as those with at least 5 years of presence in the data (without asthma or with non-severe asthma) before their first year of severe asthma.

We used Poisson regression with over-dispersion to quantify the annual rate of change, in relative percentage terms from the previous year, of outcomes over the above-mentioned time axes. The counts of variables of interest as the dependent variables and time axis of interest as the independent variable (and with logarithm of number of patients per time unit as the offset variable). Trends were quantified for each time axis with the time variable of interest as the only independent variable. However, given the potential non-linear trends of asthma severity across age [27–29], for the analysis of age trends we added the quadratic term of age (age^2) as a secondary independent variable. The results of such trends were expressed in relative terms. For example, a change in the prevalence of a variable in the sample from 20 to 10% would correspond to a relative 50% decline.

In addition, to separate the potential influence of identifiable factors associated with maintenance or episodic OCS use from secular trends, we fitted adjusted generalised linear models (with logistic distribution and logit function for chronic use, and Poisson distribution with over-dispersion and logarithmic link function for episodic use). In addition to the calendar year as the independent variable of interest, these models were adjusted for sex, age (at the beginning of each follow-up period), socio-economic status (defined as the neighbourhood income quintile for the index year), reliever medication use (defined as the number of canisters of SABA dispensed in the previous period), intensity of inhaled corticosteroids (ICS) use (measured in terms of Medication Possession Ratio [MPR] in the previous period: the number of days covered with ICS prescriptions divided by the total number of days in the period), asthma-related inpatient admissions and asthma-related outpatient encounters (by specialty type) in the previous year, as well as the presence of coexisting conditions. The latter was defined as 17 binary indicators of chronic conditions constituting the Charlson comorbidity index based on inpatient and outpatient diagnostic codes (after removing asthma-related codes) during the same follow-up period [30]. We performed a sensitivity analysis by repeating the analyses after removing the individuals who had other respiratory conditions.

We considered p-values (p) significant at 0.05 level (two-tailed). We used SAS Enterprise (version 7.15) to conduct the analyses.

## Results

A total of 201,289 patients met the case definition of asthma. Of these, 4.5% of entered the cohort by satisfying the inpatient component of case definition, 76.1% by satisfying the outpatient component, and 19.4% by satisfying the medication component. Among the initial cohort, 21,144 (mean age 28.7, 55.4% female) had at least one follow-up period compatible with severe asthma, constituting the study sample. These individuals contributed 40,803 patient-years of data for this study. Table 1 provides the basic demographic characteristics of the cohort. The average number of follow-up periods was 8.75 and on average 1.93 periods for each patient (22.0% of total follow-up time) were labelled as severe asthma.

**Table 1:** Baseline characteristics of the sample

Variable	Value
Total sample	21,144
Female; N (%)	11,722 (55.4%)
Age at index date; Mean (SD)	28.7 (9.4)
Socio-economic status; N (%)	
Quintile 1	4624 (21.9%)
Quintile 2	4030 (19.1%)
Quintile 3	4077 (19.3%)
Quintile 4	4073 (19.2%)
Quintile 5	3916 (18.5%)
Unknown	424 (2%)
Variables estimated during follow-up	
Patient years with severe asthma	40,803
Patient years associated with maintenance OCS use	3348 (8.2%)
Episodic OCS use; mean number of episodes (SD)	0.89 (0.79)
Patient-years with at least one episodic use of OCS; N (%)	30,684 (75%)
Patient-years with asthma-related hospital admission; N (%)	1502 (3.7%)
Patient-years with asthma-related physician visits; N (%)	
GP visits	25,425 (62.3%)
Respirologist	1983 (4.9%)
General internist	1012 (2.5%)
SABA canisters used per patient-year, Mean (SD)	6.2 (5.4)
Medication Possession Ratio for ICS per patient-year; mean (SD)	26.7% (29.4%)
Comorbidity: N(%)	
Group 1 (Myocardial infarction)	90 (0.2%)
Group 2 (Congestive heart failure)	369 (0.9%)
Group 3 (Peripheral vascular disease)	169 (0.4%)
Group 4 (Cerebrovascular disease)	173 (0.4%)
Group 5 (Dementia)	84 (0.2%)
Group 6 (Chronic pulmonary disease)	7818 (19.2%)
Group 7 (Connective tissue disease)	641 (1.6%)
Group 8 (Peptic ulcer disease)	308 (0.8%)
Group 9 (Mild liver disease)	581 (1.4%)
Group 10 (Diabetes without complication)	2105 (5.2%)
Group 11 (Diabetes with complication)	130 (0.3%)
Group 12 (Paraplegia and Hemiplegia)	81 (0.2%)
Group 13 (Renal disease)	276 (0.7%)
Group 14 (Cancer)	435 (1.1%)
Group 15 (Moderate or Severe liver Disease)	67 (0.2%)
Group 16 (Metastatic Carcinoma)	78 (0.2%)
Group 17 (AIDS/HIV)	162 (0.4%)

*OCS* oral corticosteroid, *ICS* inhaled corticosteroids, *SABA* short-acting beta agonists, *SD* standard deviation

The average prednisone-equivalent dose of OCS in patient-years labelled as severe asthma was 382.5 mg per patient-year (1.05 mg of prednisone per day). Overall, OCS use of any type declined by a relative 1.8% per year during the study period (P < 0.001).

### Trends in maintenance OCS use

**Fig. 1:**
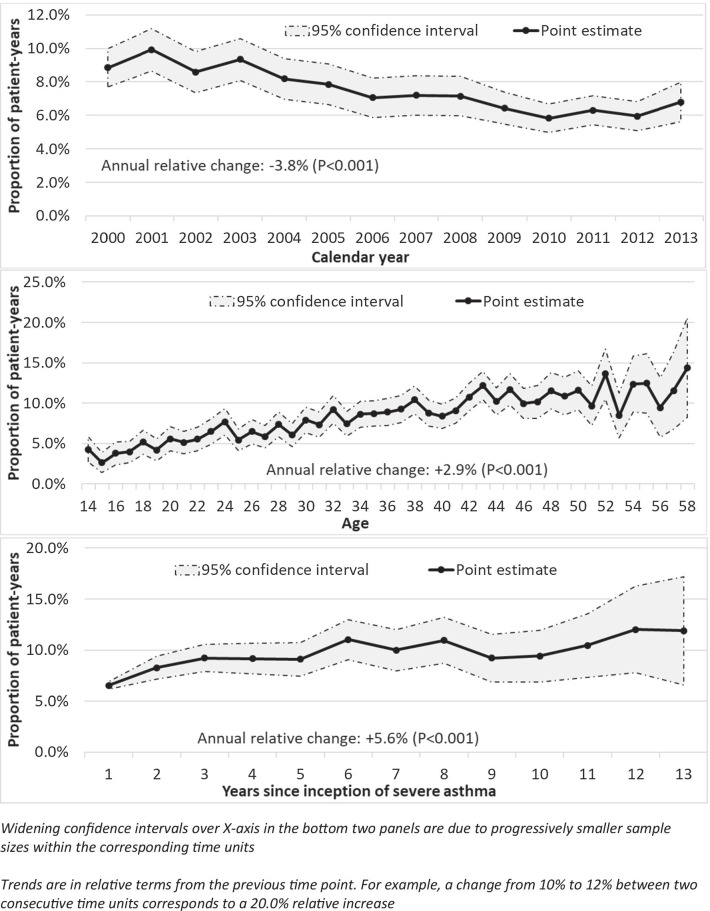
Trends of maintenance oral corticosteroid use over calendar time (top panel), years of age (middle panel), and years since onset of severe asthma (bottom panel)

In total, 8.2% (3,348) of follow-up periods were associated with maintenance OCS use. The average prednisone-equivalent daily dose of OCS among chronic users was 6.23 mg. Figure 1 depicts the trends of maintenance OCS use over time, age, and duration of severe asthma.

The average proportion of patient-years with severe asthma associated with maintenance OCS use during the first three calendar years (2000–2002) was 9.1% (Fig. 1—top panel). The corresponding value for the last three years (2011–2013) was 6.3%. On average, maintenance OCS use declined by a relative 3.8%/year (p < 0.001).

In contrast, the trend of maintenance OCS use over years of age showed an increase (Fig. 1—middle panel). Among the youngest five groups (14–18 years of age), an average of 4.0% of patients were maintenance OCS users, which increased to 12.1% among the oldest five groups (54–58 years of age). On average, maintenance OCS use increased by a relative 2.9% per one-year increase in age (p < 0.001). However, there was a non-linear component (coefficient for age^2 of − 0.0007, p < 0.001), indicating that the increasing trend flattened over time. For example, the model-estimated increase in maintenance OCS use from age 14 to 15 was 6.3%, from 29 to 30 was 4.1%, and from 56 to 57 was only 0.1%.

The trend over the time-course of severe asthma was also upward (Fig. 1—bottom panel). In the first three years of severe asthma, 8.0% of patient-years were associated with maintenance OCS use, which increased to 11.4% in the last three years (years 11–13 since the onset of severe asthma). On average, with each year since severe asthma onset, the proportion of maintenance OCS users increased by a relative 5.6% (P < 0.001).

### Trends in episodic OCS use

**Fig. 2:**
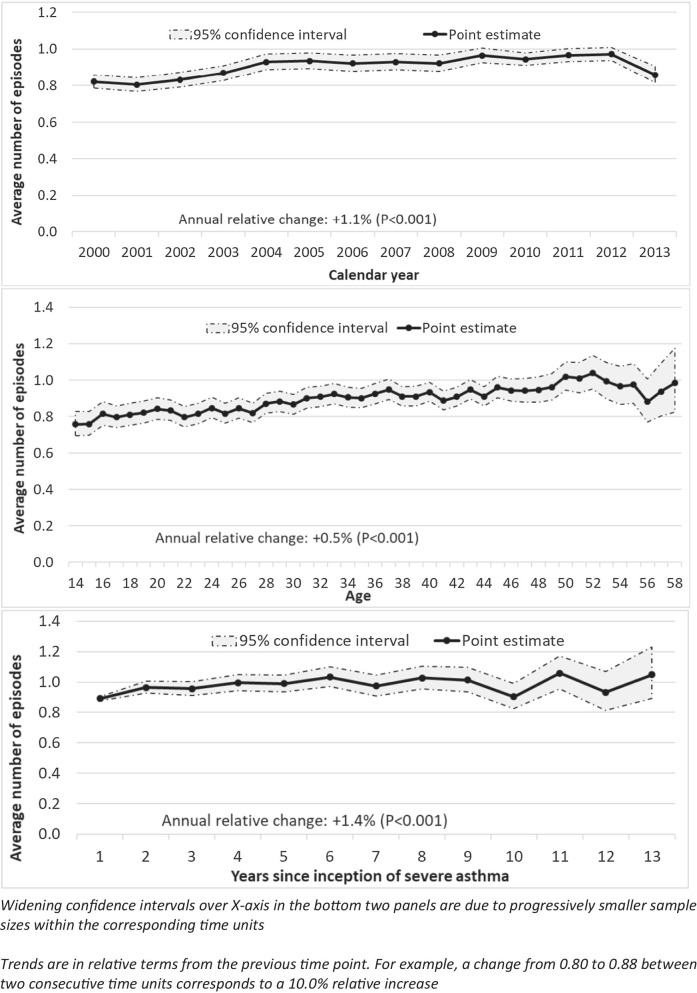
Trends of episodic corticosteroid use over calendar time (top panel), age (middle panel), and years since incidence of severe asthma (bottom panel)

In total, 75.2% (n = 30,684) of follow-up periods were associated with at least one episode of OCS use. The average number of OCS episodes per follow-up period was 0.89 (SD 0.79). Figure 2 depicts the trends of episodic OCS use over the three time axes of interest (calendar time, years of age, and years since the onset of severe asthma.)

Episodic OCS use demonstrated a slightly upward trend over calendar time (Fig. 2—top panel). In the first three years (2000–2002), the average number of episodes of OCS use was 0.82/year, whereas in the last three years (2011–2013) it was 0.93/year (with a clear decline in the last year of observation). On average, episodic OCS use increased by a relative 1.1%/year (P < 0.001).

The trend over years of age was also increasing (Fig. 2—middle panel). Among the youngest five groups (14–18 years of age), the average number of episodes of OCS use was 0.79/year, increasing to 0.95/year among the oldest five age groups (54–58 years of age). On average, there was a relative increase of 0.5% (P < 0.001) in episodic OCS use for every one year increase in age. Unlike for maintenance use, no non-linearity for the age trend was identified (p for age^2 0.068).

The trend over time-course of severe asthma was also upward (Fig. 2—bottom panel). In the first three years of severe asthma, the average number of episodes of OCS use was 0.94, which increased to 1.01 in the last three years of available data (years 11–13). Within the time-course of severe asthma, the average number of episodes of OCS use increased by a relative 1.4%/year (P < 0.001).

### Factors associated with time trends of maintenance and episodic OCS use

Table 2 provides the results of the regression analyses for maintenance and episodic OCS use. Female sex and older age were associated with a higher likelihood of both maintenance and episodic OCS use, while higher socio-economic status was associated with a higher likelihood of maintenance but not episodic OCS use. Higher intensity of ICS use in the previous period was associated with higher maintenance and episodic use of OCS, most likely reflecting the severity of asthma. This was also the case for reliever medication use. Similarly, asthma-related inpatient and outpatient encounters with general practitioners (versus no encounters) were associated with higher likelihood of maintenance and episodic OCS use in the next year. Outpatient encounters with specialists (internists and respirologists, versus no encounters with any specialist) were associated with higher likelihood of maintenance use, but such associations for episodic use were not statistically significant. Several comorbid conditions were associated with chronic use, and fewer with episodic use. After controlling for covariates, the annual relative rate of decline in maintenance use was 2.8% and the annual relative increase in episodic use was 0.9%; both remained statistically significant.

**Table 2:** Factors associated with maintenance and episodic oral corticosteroid use

Parameter	Maintenance use			Episodic use		
	OR	95%CI	P	RR	95%CI	P
Female sex	1.11	1.00–1.23	0.05*	1.08	1.05–1.11	< 0.01*
SES quintile	1.04	1.01–1.07	0.02*	0.99	0.99–1.00	0.09
Calendar year	0.97	0.96–0.98	< 0.01*	1.01	1.01–1.01	< 0.01*
Age	1.31	1.25–1.38	< 0.01*	1.02	1.01–1.03	< 0.01*
ICS Medication Possession Ratio^#^	1.38	1.19–1.60	< 0.01*	1.11	1.07–1.15	< 0.01*
Number of dispensed SABA canisters^#^	1.03	1.02–1.03	< 0.01*	1.01	1.00–1.01	< 0.01*
Asthma-related hospital admission^#^	2.90	2.48–3.40	< 0.01*	1.15	1.12–1.20	< 0.01*
Asthma-related specialist visit^#^	2.86	2.47–3.29	< 0.01*	1.05	0.99–1.12	0.09
Asthma-related internist visit^#^	1.58	1.31—1.92	< 0.01*	1.06	0.97–1.15	0.17
Asthma-related general practitioner visit^#^	1.26	1.14–1.40	< 0.01*	1.08	1.06–1.10	< 0.01*
Comorbidity						
Group 1 (Myocardial infarction)	0.96	0.51–1.81	0.91	1.07	0.87–1.32	0.53
Group 2 (Congestive heart failure)	1.34	0.92–1.97	0.13	0.96	0.86–1.08	0.49
Group 3 (Peripheral vascular disease)	1.57	0.98–2.51	0.06	0.91	0.80–1.03	0.13
Group 4 (Cerebrovascular disease)	1.04	0.63–1.70	0.88	1.01	0.88–1.17	0.85
Group 5 (Dementia)	0.91	0.40–2.09	0.83	1.08	0.85–1.38	0.53
Group 6 (Chronic pulmonary disease)	1.58	1.43–1.74	< 0.01*	1.17	1.13–1.20	< 0.01*
Group 7 (Connective tissue disease)	1.67	1.27–2.21	< 0.01*	0.91	0.83–0.98	0.01*
Group 8 (Peptic ulcer disease)	0.84	0.54–1.31	0.44	1.02	0.91–1.14	0.74
Group 9 (Mild liver disease)	0.80	0.57–1.11	0.18	1.00	0.92–1.08	0.98
Group 10 (Diabetes without complication)	1.22	1.03–1.44	0.02*	1.03	0.97–1.08	0.36
Group 11 (Diabetes with complication)	0.99	0.74–1.32	0.94	1.04	0.94–1.14	0.43
Group 12 (Paraplegia and Hemiplegia)	1.06	0.73–1.54	0.77	1.05	0.88–1.26	0.58
Group 13 (Renal disease)	1.59	1.29–1.96	< 0.01*	0.97	0.91–1.05	0.48
Group 14 (Cancer)	1.27	1.08–1.49	< 0.01*	1.00	0.95–1.05	0.91
Group 15 (Moderate or Severe liver Disease)	0.92	0.68–1.26	0.61	1.01	0.95–1.07	0.74
Group 16 (Metastatic Carcinoma)	1.56	1.26–1.92	< 0.01*	0.93	0.86–1.01	0.08
Group 17 (AIDS/HIV)	0.99	0.89–1.09	0.84	0.99	0.96–1.01	0.54

*ICS* Inhaled corticosteroids, *SABA* Short-acting beta-agonist, *SES* Socio-economic status, *OR* odds ratio, *RR* relative rate

*Significant at 0.05 level

^#^These variables were measured during the preceding period to avoid reverse causality bias in estimates of associations

### Sensitivity analysis

When we removed 19.2% of patients with other chronic respiratory conditions, results largely remained the same. The annual relative rate of change for maintenance OCS use was as follows: -4.7% per calendar year, 2.7% per year of age, and 4.3% per year since the inception of asthma. For episodic OCS use, the patterns were as follows: 1.0% per calendar year, 0.4% per year of age, and 0.1% per year since the inception of asthma.

## Discussion

By analysing healthcare resource use data of the entire population of a well-defined geographic region, we have documented the trends of maintenance and episodic use of oral corticosteroids over calendar time, age, and years since the onset of severe asthma. Maintenance OCS use declined over calendar time, from 9.12% in the first three years (2000–2002) to 6.35% in the last three years (2011–2013). This was partially countered by increasing use of OCS episodically, averaging from 0.82 episodes per patient-year in the first three years to 0.93 episodes per patient-year in the last three years of the study. The overall pattern of OCS use of any type, however, was consistent with a decline of 1.8% per year during the study period. Both maintenance and episodic use increased steadily over the age and over the time-course of severe asthma.

The observed trends largely persisted after controlling for sociodemographic characteristics, asthma-related medication use, asthma-related health services use, and burden of comorbidity. As such, the decline in maintenance use cannot be attributed to the increasing use of inhaled corticosteroids (especially in combination with long-acting beta-agonists) over time, as documented in this population [30]. As well, one explanation for this observation could have been the increased risk of comorbidities that might require OCS use in the older age groups. However, the trends persisted after controlling for comorbidity and age. The increasing awareness of the long-term side effects of OCS might have resulted in a decline in care providers’ recommendation for, and patients’ acceptance of, maintenance OCS therapies; such a decline in maintenance use might put some patients at increased risk of exacerbations that result in episodic use of OCS. However, the increasing episodic OCS use over time is in conflict with the downward trend of asthma-related admissions in the same study population, as documented elsewhere [30]. This in itself can be explained by the notion that increasing (and earlier) use of OCS in exacerbation episodes might avoid the escalation of impairment to the point of requiring inpatient care. Overall, changes in practice patterns due to updated guidelines [31], or changes in patients’ and care providers’ perception about OCS use might have played a role in the observed trends, but such information is not captured in administrative data. On the other hand, the upward trends (for both maintenance and episodic use) over age and time-course of severe asthma could reflect the gradual worsening of asthma over time, as supported by existing evidence [31].

To the best of our knowledge, ours is one of the first studies that have evaluated the trends in OCS use in severe asthma. A few investigators have provided evidence on the extent of systemic corticosteroid use. Zeiger et al. reported a prevalence of 8.2% for maintenance OCS among 9,546 patients aged 18 to 64 years with persistent asthma in a large managed care organisation in the United States (US) [9], which is similar to our estimates. On the contrary, Wysocki, et al. reported a prevalence of 30.4% of chronic systemic corticosteroid use in 319 patients with objectively verified severe asthma as part of a national registry program in the US [14]. There could be several reasons behind the difference between Wysocki et al. results and numbers reported by Zeiger et al. and in our study. The inclusion of non-oral systemic corticosteroids use, self-reported assessment of intake, potential self-selection of patients into a severe asthma registry, and the definition of severe asthma (which was assessed in clinical settings), could account for this discrepancy.

The main strength of this study was the use of population-based data, which cover the entire population of a well-defined geographic region. Access to > 40,000 patient-years of data associated with severe asthma enabled robust statistical inference while 14 years of follow-up enabled estimation of long-term trends. Also, we defined severe asthma based on a validated algorithm specifically developed for Canadian administrative health data [21]. The limitations of our study should also be acknowledged. First, filled prescriptions do not necessarily reflect actual drug usage. However, there is no strong reason to suspect that any discrepancy between filled prescriptions and actual intake would substantially change over time; therefore, such discrepancy is unlikely to threaten the validity of the analyses of the trends. Also, as our intention was to report the overall trends in a population-based sample of severe asthma patients, we applied minimal exclusion criteria, in particular not excluding patients with other chronic conditions that are associated with OCS use. It is not clear to what extent OCS use was attributed to asthma in comparison to other conditions. The persistence of observed time trends in the adjusted analysis that included comorbid conditions suggests that the trends in OCS use can indeed be attributed to asthma-related prescriptions. Further, some other factors that might explain part of the observed trend in the OCS use pattern (including smoking) were not available in the administrative health data. Finally, our study only included patients up to 58 years of age. The age criterion was imposed as we were concerned about the inclusion of patients with Chronic Obstructive Pulmonary Disease (COPD). This comes at the cost of older patients with severe asthma being under-represented in our sample. Similarly, this sample did not include paediatric asthma patients. There is a reversal in the epidemiology of asthma between boys and girls as they age [28]. Exploring the effect of such change on pattern of OCS use in children should be pursued in future studies.

Our study also raises several questions. The effect of the arrival of multiple biologics with corticosteroid-sparing effects on these trends should be monitored. The declining trends in maintenance OCS use was documented for the period before any biologics were prescribed (and in general, as the use of omalizumab, the only biologic in the data, was very low during the study period). As such, future decline in OCS use will not necessarily be due to the uptake in the use of biologics. Further, given that the included patient and disease characteristics available in administrative health data could not account for the observed trends, future studies should explore the role of other factors such as changes in guidelines for the management of severe asthma, patients’ and care providers’ adherence to such guidelines, or changes in the perception of benefits and risks associated with OCS use among patients and care providers. The effect of environmental changes (e.g., trends in urbanisation, occupational exposure, climate change) should also be considered. In particular, future studies can juxtapose temperature and air pollution data against trends in asthma-related medications and health services use to provide further insight into the potentially significant effect of seasonality and climate change on these outcomes. We also note that our results pertain to a jurisdiction with a publicly funded health care system. The generalisability of the results to other jurisdictions is not certain, especially given the different guideline-based treatment algorithms and policies for access to asthma medications. Finally, whether the risk of adverse outcomes is different between maintenance and episodic OCS use has not been evaluated. As well, the comparative systemic effects of OCS and high-dose ICS in this patient population has only been partially studied [32].

## Conclusion

A recent large study among the general population has shown that even one course of OCS is associated with an increased risk of major adverse events [33]. As a result, systemic corticosteroids are among the most common causes of drug-related complications [34]. A recent study from Sweden has demonstrated substantial economic and health care burden for asthma patients on maintenance OCS treatment [35]. While the overall use of OCS, and maintenance OCS use, have declined in our study sample, the episodic OCS use has increased over time. Such trends could only be partially explained by the observable socio-demographic characteristics, extent of asthma medication use, asthma-related inpatient and outpatient encounters, and burden of comorbidity. OCS use was particularly high among older patients and patients with long-lasting severe asthma. Given such a dynamic landscape, future studies should closely monitor the pattern of systemic corticosteroid use especially with the increasing availability of several existing and novel therapies for severe asthma.

## Supplementary Information

**Additional file 1: Table S1.** List of asthma-related medications.

## Data Availability

The data that support the findings of this study are available from British Columbia’s Ministry of Health but restrictions apply to the availability of these data, which were used under license for the current study, and so are not publicly available. The data are protected under the provincial Freedom of Information and Protection of Privacy Act, which stipulated that the data can only be accessed from within Canada. Access to the data will require formal permission by the Data Stewards within the Ministry of Health.

## Abbreviations

BC: British Columbia
ICD: International Classification of Diseases
ICS: Inhaled corticosteroids
OCS: Oral corticosteroids
OR: Odds ratio
RR: Relative rate
SABA: Short-acting beta agonists
SD: Standard deviation
MPR: Medication Possession Ratio
p: P value
