# Supplementary material for: Trends in oral corticosteroids use in severe asthma: a 14-year population-based study
Source: Respir Res. 2021 Apr 9;22:103. doi: 10.1186/s12931-021-01696-x (PMC8034163; doi:10.1186/s12931-021-01696-x)
Supplement: Supplementary file 1 — Additional file 1: Table S1. List of asthma-related medications. [file 12931_2021_1696_MOESM1_ESM.docx]

| Category | Drug Identifier Number |
| --- | --- |
| Inhaled corticosteroids | 00374407, 00828521, 00828548, 00851752, 00851760, 00852074, 00872334, 00893633, 00897353, 01949993, 01950002, 01978918, 01978926, 02079976, 02174731, 02174758, 02174766, 02174774, 02213583, 02213591, 02213605, 02213613, 02213710, 02213729, 02215039, 02215047, 02215055, 02216531, 02229099, 02237244, 02237245, 02237246, 02237247, 02242029, 02242030, 02244291, 02244292, 02244293, 02285606, 02285614, 02303671 |
| Leukotriene receptor antagonists | 02236606, 02238216, 02238217, 02243602, 02247997 |
| Inhaled long-acting beta agonists | 02136139, 02136147, 02211742, 02214261, 02230898, 02231129, 02237224, 02237225 |
| Inhaled short-acting beta agonists | 00249920, 00620955, 00620963, 00790419, 00812463, 00832758, 00832766, 00851841, 00867179, 00874086, 01932691, 01938851, 01938878, 02046741, 02048760, 02063689, 02084333, 02091186, 02148617, 02152568, 02164434, 02164442, 02165368, 02165376, 02192675, 02212315, 02212323, 02213451, 02213478, 02214997, 02215004, 02215616, 02215624, 02215632, 02216949, 02229862, 02231430, 02231488, 02231678, 02231783, 02231784, 02232570, 02236931, 02236932, 02236933, 02239365, 02241497, 02243115, 02243828, 02244914, 02245669, 02259583, 02261324, 02326450 |
| Cromolyns | 00261238, 00534609, 00555649, 00638641, 02046113, 02049082, 02219468, 02231431, 02231671 |
| Inhaled corticosteroid and long-acting beta agonists | 02240835, 02240836, 02240837, 02245126, 02245127, 02245385, 02245386 |
| Omalizumab | 02260565 |
| Short-acting beta agonists (oral) | 00003891, 00786616, 00860808, 00894249, 00894257, 00897345, 01926934, 01945203, 01947222, 01986864, 02022125, 02035421, 02069571, 02146843, 02146851, 02154412, 02173360, 02208229, 02208237, 02208245, 02212390, 02213400, 02213419, 02213427, 02213435, 02213443, 02213486, 02232987, 02236783, 02239366 |

Appendix Table A1: List of asthma-related medications
